# RP-HPLC Method Development and Validation of Synthesized Codrug in Combination with Indomethacin, Paracetamol, and Famotidine

**DOI:** 10.1155/2020/1894907

**Published:** 2020-07-01

**Authors:** Mohyeddin Assali, Murad Abualhasan, Nihal Zohud, Noura Ghazal

**Affiliations:** Department of Pharmacy, Faculty of Medicine and Health Sciences, An-Najah National University, P.O. Box 7, Nablus, State of Palestine

## Abstract

**Background:**

Indomethacin is considered a potent nonsteroidal anti-inflammatory drug that could be combined with Paracetamol to have superior and synergist activity to manage pain and inflammation. To reduce the gastric side effect, they could be combined with Famotidine. *Methodology*. A codrug of Indomethacin and Paracetamol was synthesized and combined in solution with Famotidine. The quantification of the pharmaceutically active ingredients is pivotal in the development of pharmaceutical formulations. Therefore, a novel reverse-phase high-performance liquid chromatography (RP-HPLC) method was developed and validated according to the International Council for Harmonization (ICH) Q2R1 guidelines. A reverse phase C18 column with a mobile phase acetonitrile: sodium acetate buffer 60 : 40 at a flow rate of 1.4 mL/min and pH 5 was utilized.

**Results:**

The developed method showed good separation of the four tested drugs with a linear range of 0.01–0.1 mg/mL (*R*^2^ > 0.99). The LODs for FAM, PAR, IND, and codrug were 3.076 × 10^−9^, 3.868 × 10^−10^, 1.066 × 10^−9^, and 4.402 × 10^−9^ mg/mL respectively. While the LOQs were 9.322 × 10^−9^, 1.172 × 10^−10^, 3.232 × 10^−9^, and 1.334 × 10^−8^ mg/mL, respectively. Furthermore, the method was precise, accurate, selective, and robust with values of relative standard deviation (RSD) less than 2%. Moreover, the developed method was applied to study the *in vitro* hydrolysis and conversion of codrug into Indomethacin and Paracetamol.

**Conclusion:**

The codrug of Indomethacin and Paracetamol was successfully synthesized for the first time. Moreover, the developed analytical method, to our knowledge, is the first of its kind to simultaneously quantify four solutions containing the following active ingredients of codrug, Indomethacin, Paracetamol, and Famotidine mixture with added pharmaceutical inactive ingredients in one HPLC run.

## 1. Introduction

Nonsteroidal anti-inflammatory drugs (NSAIDs) are among the most consumed and prescribed drugs for both pain and inflammation worldwide [1]. Their blockage of prostaglandin synthesis by inhibiting cyclooxygenase (COX) is responsible for both the desired anti-inflammatory effects and the undesired gastrointestinal effects [2–4]. Based on COX selectivity, NSAIDs are divided into two families: nonselective NSAIDs that block both cyclooxygenase I & II and selective cyclooxygenase II inhibitors [5–7].

Indomethacin (IND) is an example of a potent nonselective COX inhibitor that showed efficient analgesia with antipyretic and anti-inflammatory activities [8]. It is classified as an indole-acetic acid derivative according to the NSAIDs chemical classification with the chemical name of 1-(*p*-chlorobenzoyl)-5-methoxy-2-methylindole-3-acetic acid [9]. It is a poorly soluble class II compound with a half-life of 4-5 h [10, 11]. It is utilized to treat rheumatoid diseases by elevating the inflammation. Moreover, it can decrease the risk of colon cancer by providing chemoprotective effects against tumors [12, 13]. Like other NSAIDs, it appeared to have gastrointestinal, renal, and other side effects [14]. The gastric side effect could be reversed by the administration of Famotidine (FAM), which is a competitive histamine H2-receptor antagonist that inhibits the secretion of gastric acid and also increases collagen secretions [15, 16].

Recently, there has been a trend for combining NSAIDs with Paracetamol (PAR) as this often provides a synergic analgesic effect and reduces the adverse effects resulting from NSAIDs [17]. Paracetamol, *N*-acetyl-*p*-aminophenol (also known as acetaminophen), is utilized globally as an analgesic and antipyretic drug. Regarding the mechanism of action which is considered to be a weak inhibitor of the synthesis of prostaglandins (PGs), their effects *in vivo* are similar to these COX-2 inhibitors [18]. Although their analgesic effects are often weaker than NSAIDs, it has better tolerance, and accordingly, it is often preferred [19].

Indomethacin is considered strong and potent anti-inflammatory activity against rheumatoid arthritis and other inflammatory diseases and Paracetamol is considered as the first-choice medication for both acute and chronic pain [20]. Therefore, the combination of Indomethacin with Paracetamol provides excellent anti-inflammatory and analgesic activities with a reduction of the Indomethacin side effects. Seidman and Melander reported equianalgesic activity with milder side effects upon the administration of Paracetamol with a low dose of Indomethacin in comparison to the high dose of Indomethacin alone for the treatment of rheumatoid arthritis [21]. Famotidine is considered the most potent H2 antagonist for the treatment of peptic ulcers and was found to be effective for prevention of Indomethacin-induced gastric injury even in the lowest dose [22, 23]. Therefore, we aim to synthesize a novel codrug of Indomethacin and Paracetamol (IND-PAR) through a hydrolyzable ester bond combined in solution with Famotidine.

Reverse phase-high performance chromatography (RP-HPLC) is considered one of the most common analytical techniques used for the development and characterization of pharmaceutical products [24, 25]. Moreover, HPLC provides a rapid, sensitive, and precise technique to separate and identify the analyzed drugs in combination or the used pharmaceutical dosage forms. Therefore, It is necessary to validate the developed HPLC method according to the International Council for Harmonization (ICH) and the United States Pharmacopeia (USP) requirements [26, 27].

Moreover, a simple and universal RP-HPLC method of analysis was developed and validated for the successful separation of a mixture containing four components: codrug, Indomethacin, Paracetamol, and Famotidine in the formulation. The developed method was used to study the hydrolysis profile of the codrug in the presence of the esterase enzyme.

## 2. Materials and Methods

### 2.1. Materials and Reagents

Indomethacin (IND), Famotidine (FAM), 4-(Dimethylamino) pyridine (DMAP), silica gel, and N-(3-Dimethylaminopropyl)-N′-ethylcarbodiimide hydrochloride (EDC) 98% were purchased from Sigma-Aldrich Company. Paracetamol (PAR) was purchased from Sun Pharma Ltd. (Nablus, Palestine). Sodium acetate trihydrate, disodium hydrogen phosphate, potassium hydrogen phosphate, ethyl acetate 99.5% (EtOAc), hexane (Hex), and dichloromethane (DCM) were purchased from CS Company, Haifa. Acetonitrile supragradient grade for chromatography (ACN) and triethylamine (Et_3_N) were purchased from SDFCL. Porcine liver esterase (PLE) was purchased from Sigma-Aldrich, USA. Inactive pharmaceutical ingredients: microcrystalline cellulose, magnesium stearate, aerosol, and Ac-Di-Sol were donated by Jerusalem Pharmaceuticals Company, Palestine.

### 2.2. Instrumentations

High-Performance liquid chromatography (Waters 1525, Singapore) binary HPLC pump and waters 2298 photodiode Array Detector were used. Nuclear Magnetic Resonance (NMR) spectrum was recorded on Bruker 500 MHz–Avance III, Switzerland. The high-resolution mass spectrum (HRMS) was recorded on a Shimadzu LCMS-IT-TOF utilizing ESI (+) method.

### 2.3. Synthesis of Indomethacin-Paracetamol (IND-PAR) Codrug

Dichloromethane (8 mL) was added to a mixture of Indomethacin (200 mg, 0.60 mmol), Paracetamol (101.4 mg, 0.67 mmol), EDC (128.6 mg, 0.67 mmol), and DMAP (75.1 mg, 0.62 mmole) and was stirred at room temperature overnight under argon. The reaction was treated with DCM and 1 M HCl three times. The collected organic layers were evaporated using a rotary evaporator. Then the crude product was purified using flash chromatography on silica gel eluted with a mobile phase of Hex: EtOAc 1 : 2 to provide a yellow solid product with a yield 70% (220 mg) and *R*_*f*_ = 0.63 (Hex: EtOAc 1 : 2). ^1^H NMR (500 MHz, CDCl_3_): *δ* 2.11 (s, 3H, COCH_3_), 2.42 (s, 3H, CH_3_ indole), 3.81 (s, 3H, OCH_3_), 3.86 (s, 2H, CH_2_CO), 6.68 (dd, 1H, *J* = 9.2 Hz, *J* = 2.3 Hz, H-7 indole), 6.68 (d, 1H, *J* = 9.2 Hz, H-9 indole), 6.98 (d, 2H, *J* = 8.8 Hz, phenyl), 7.02 (d, 1H, *J* = 2.3 Hz, H-6 indole), 7.45 (dd, 4H, *J* = 8.8 Hz, *J* = 1.9 Hz, phenyl), 7.65 (d, 2H, *J* = 8.4 Hz, phenyl). HRMS (ESI, m/z): calcd. for C_27_H_24_N_2_O_5_Cl [M +  H]^+^ 491.1374, found 491.1372.

### 2.4. HPLC Analytical Method Development

#### 2.4.1. Prepared Solutions

*(1) Buffer Solution pH 6*. 13.6 g of sodium acetate trihydrate was dissolved in 750 mL HPLC water, then 1 mL of Et_3_N was added, diluted with HPLC water to 1 L, and adjusted to pH 6.0 with glacial acetic acid. The mobile phase was firstly prepared using a mixture of sodium acetate Buffer:ACN, 93 : 7 [28].

*(2) Diluent 1*. 6.8 g of potassium dihydrogen phosphate was dissolved in 1 L HPLC water and adjusted to a pH 6.0 using glacial acetic acid.

*(3) FAM, IND, PAR, and Codrug Standard Solutions*. 2.5 mg of the standard was weighed into 25 mL volumetric flask; 5 mL methanol was added and then diluted up to 25 mL by the prepared diluent.

*(4) Standard Solution Mixture*. 2.5 mg of each FAM, IND, PAR, and codrug was diluted with HPLC acetonitrile to the volume (25 mL).

#### 2.4.2. pH, Mobile Ratio, and Diluents Used in Method Development Trials

Different mobile phase composition, pH, and diluents were tried throughout the analytical method development. The used mobile phases and diluents at different pH are summarized in Table 1.

### 2.5. Analytical Method Validation

The analytical method was developed according to USP and ICHQ2R1 guidelines and validated using the following parameters: linearity, range, accuracy, precision, robustness, and ruggedness [29]. All prepared parameters were in triplicates.

#### 2.5.1. Linearity and Range

Linearity was measured by preparing a serial five concentrations in the range of 0.01–0.1 mg/mL form a preprepared stock solution of 1 mg/mL. The calibration curves were built by plotting the mean area under the curve (AUC) obtained from the HPLC against concentrations. The regression equation and the squared correlation coefficient (*R*^2^) were calculated for each ingredient curve.

#### 2.5.2. Accuracy

Accuracy and selectivity validation parameters were calculated by preparing a standard solution of a mixture of four drugs, having a concentration of 0.24 mg/mL for each drug. Three concentration levels of 80%, 100%, and 120% of the standard concentration were made. The three solutions were prepared containing different excipients: microcrystalline cellulose, magnesium stearate, aerosol, and Ac-Di-Sol. The accuracy was evaluated by calculating the percentage of recovery.

#### 2.5.3. Selectivity

The selectivity of the developed method was examined as the eluted peaks are well separated and not affected by any of the added excipients.

#### 2.5.4. Precision

Precision was performed at different levels. At first, instrument precision was done by injecting the standard mixture 9 times; the % RSD of the generated peaks of the chromatogram was calculated. An intermediate precision including interday and between analyst precision was examined on 0.08 mg/mL and 0.1 mg/mL concentration, respectively. The percentage relative standard deviation was calculated for both mixtures.

#### 2.5.5. Robustness

The robustness of the developed method was performed by doing minor modifications on the method parameters, including detection wavelength, different mobile phase pH, and flow rate [30]. The studied robustness parameters were the pH effect of the mobile phase (4.9, 5.0, and 5.1), the detection wavelength (273, 275, and 277 nm), and the effect of the mobile phase flow rate (1.2, and 1.4 mL/min).

#### 2.5.6. Detection and Quantification Limit (LOD & LOQ)

Limit of detection (LOD) and limit of quantification (LOQ) is an indication of the analytical method sensitivity. Signal to noise ratio in the HPLC chromatogram was used to calculate these two parameters for each compound. The LOD and LOQ value of the compound was determined when the signal to noise ratio is 3 : 1 and 1 : 10, respectively.

### 2.6. Hydrolysis of Codrug

The synthesized codrug was incubated with an esterase enzyme to study its hydrolysis to its parent drugs (IND & PAR). This was achieved by incubating 1 mg of codrug into 10 mL phosphate buffer saline solution (pH 7.4) containing 1 mg of esterase enzyme (10 U) at 37°C for 1 h [31–33]. At different time intervals, aliquots of 1 mL were obtained, and then the concentrations were analyzed by the developed HPLC method.

### 2.7. Statistical Analysis

All prepared parameters, including the *in vitro* hydrolysis of the codrug, were performed in triplicates. The data were expressed as means ± relative standard deviation. Statistical analysis was performed on robustness parameters using the ANOVA test. Statistically, a significant difference was considered when the *p* value was <0.05.

## 3. Results and Discussion

### 3.1. Synthesis of IND-PAR Codrug

**Scheme 1:**
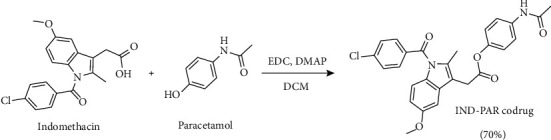
Synthetic scheme of IND-PAR codrug.

Herein, we aim to synthesize a codrug of Indomethacin and Paracetamol to obtain a synergistic analgesic, antipyretic, and anti-inflammatory activities. The synthesis of the codrug was achieved through the formation of the ester bond between IND and PAR using EDC as a coupling agent and 4-(Dimethylamino)pyridine as a base, as shown in Scheme 1. The codrug was successfully synthesized with a high yield of 70%. The structure of the codrug was confirmed by NMR and HRMS.

### 3.2. Method Development

The RP-HPLC analytical method for codrug, IND, PAR, and FAM was developed and validated according to the ICH guidelines [34]. The analytical method development was mainly based on the USP analytical method of FAM and IND and was then optimized for the best separation for the component mixture [35].

**Figure 1:**
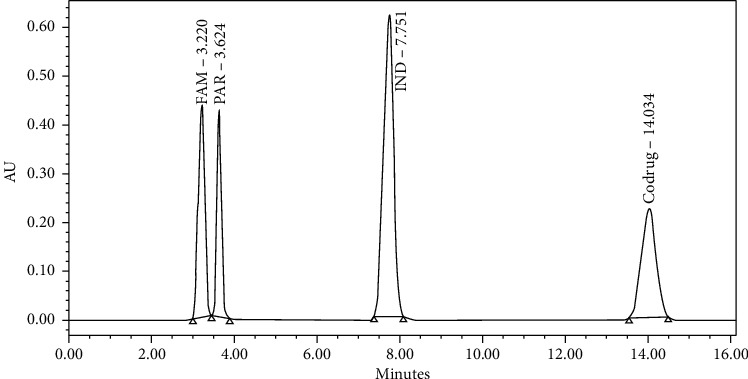
Chromatogram of the eluted peaks for the component mixture.

At first, FAM and IND mixture was injected using the USP analytical method for the Famotidine tablet. The produced HPLC chromatogram showed only one peak of FAM, while the IND was not eluted even after 40 minutes. The late elution of IND is probably due to its high lipophilicity and the high hydrophobicity of the used mobile phase (ACN: sodium acetate buffer 7 : 93). Further modification of the mobile phase pH was done in order to reduce the retention time of IND by making the mobile phase more acidic. Different pH mobile phases were tried, including 5, 5.5, and 6. The results showed that the most acidic pH mobile (pH 5) achieved early elution of IND to less than 10 min. In order to get the best separation, the mobile phase was further optimized at different solvent compositions. Lastly, the best separation was achieved at the mobile phase composition (ACN: sodium acetate buffer 60 : 40). The codrug was added to the above mixture of IND and FAM using pure ACN as the diluent. The final HPLC chromatographic conditions of the developed method were by using XTERRA® MS C18, 5 *μ*m, 4.6 × 250 mm analytical column with a flow rate of 1.4 mL/min and a detection wavelength of 275 nm. The operation temperature of the column was set at 25°C. The injection volume was 10 *μ*L and the run time was 20 minutes. The developed method showed well-separated peaks for the component mixture. The resulted peaks were symmetrical with narrow broadening eluted at different retention times: 3.220, 3.624, 7.751, and 14.034 min for FAM, PAR, IND, and codrug, respectively, as shown in Figure 1.

### 3.3. Method Validation

#### 3.3.1. Linearity and Range

The quality of an analytical method is profoundly dependent on the linearity of the calibration curve. The main characteristics of a calibration curve are the slope line, the regression, and the correlation.

**Figure 2:**
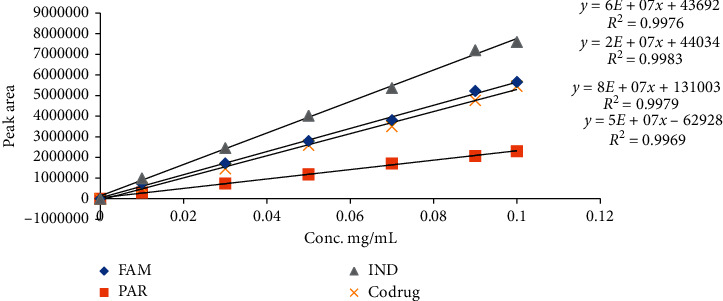
Linearity curves for compounds FAM, PAR, IND, and codrug.

The linearity of the method was measured by plotting the area under the curve obtained from the HPLC of each drug against the corresponding concentrations. The linearity was demonstrated over the concentration range (0.01–0.1 mg/mL) for FAM, PAR, IND, and codrug, respectively. The obtained goodness-of-fit (*R*^2^) was more than 0.99 that confirms the linearity between the concentration and the area under the peak. The slopes of the regression line for FAM, PAR, IND, and codrug are shown in Figure 2.

#### 3.3.2. Selectivity

The four drugs components (FAM, PAR, IND, and codrug) were formulated with the following inactive ingredients: microcrystalline cellulose, magnesium stearate, aerosol, and Ac-Di-Sol to study selectivity of the developed analytical method [36].

**Figure 3:**
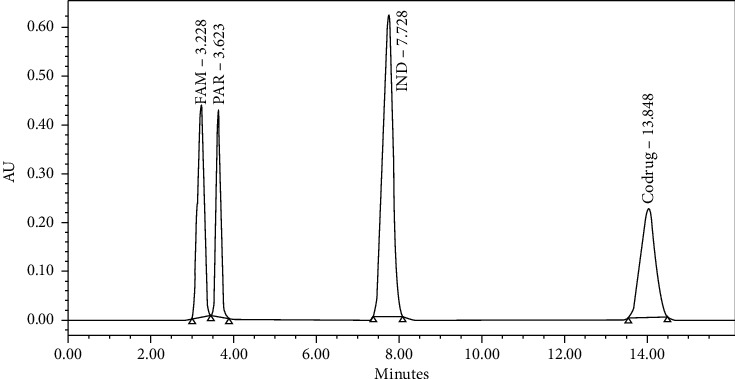
Chromatogram of the eluted peaks for the component mixture with inactive ingredients.

This parameter was investigated to show that there is no possible interference of the added tablet formula excipients on the separation and measurements of peak areas for the ingredient mixture (Figure 3).

#### 3.3.3. Accuracy

The method showed great accuracy within the tested concentration range (0.08–0.12). The percentage of RSD and percentage of recovery for all tested solutions are within the acceptable limits (100% ± 2%); the data are shown in Table 2.

#### 3.3.4. Precision

The precision of a method is the degree of agreement among individual test results when the procedure is applied repeatedly to multiple samplings.

The method precision was examined at different levels; system precision was examined by injecting 0.1 mg/mL nine times on HPLC and the % RSD was found to be less than 2.0 for all tested compounds.

The intermediate precision validation parameter at different days (intraday precision) was studied by performing three replicates measurements at two different concentrations (0.08 and 0.1 mg/mL). The results showed that the percentage relative standard deviation of the triplicate of each concentration was less than 2.0. Moreover, the repeatability was tested for different analysts by doing three replicates measurements of the mixture at 0.12 mg/mL and the result % RSD was also less than 2.0. The precision results at different precision levels are illustrated in Table 3.

#### 3.3.5. Detection and Quantification Limit (LOD & LOQ)

The detection limit or LOD is the lowest amount of analyte in a sample that can be detected but not necessarily quantified. However, the limit of quantification or LOQ is the lowest amount of analyte in a sample that can be determined quantitatively with convenient precision and accuracy. The result showed that the calculated LODs for FAM, PAR, IND, and codrug were found to be 3.076 × 10^−9^, 3.868 × 10^−10^, 1.066 × 10^−9^, and 4.402 × 10^−9^ mg/mL, respectively, while the calculated LOQs were 9.322 × 10^−9^, 1.172 × 10^−10^, 3.232 × 10^−9^, and 1.334 × 10^−8^ mg/mL, respectively.

#### 3.3.6. Robustness

Robustness is the capacity of a method to remain unaffected by minor variations in method conditions; in other words, it is a measure of the reliability of a method.

The robustness of an analytical procedure was tested by measuring its capacity of the developed method to remain unaffected by small but deliberate variations in the method parameters and providing an indication of its reliability during the normal use. For this study, the flow rate, wavelength, and pH parameters were changed for a mixture of 0.1 mg/mL. The results are summarized in Table 4. As can be observed, the % RSD values in all tested and varied parameters were less than 2.0 which indicates the good robustness of the developed analytical method. Moreover, the ANOVA test shows no significant difference for the tested compounds at different robustness validation parameters (*p* value >0.05).

#### 3.3.7. System Suitability

**Figure 4:**
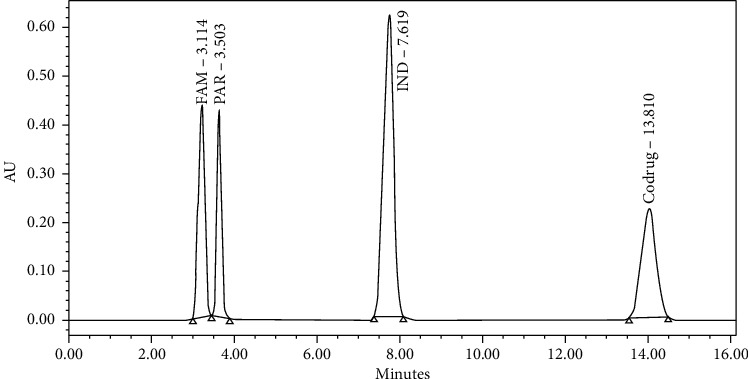
System suitability.

System suitability tests are utilized to justify that a system is performing sufficiently to guarantee confidence in the analytical method and the obtained results. The developed method showed that all of the standard system suitability parameters, including the resolution (*R*), the symmetry of the peaks theoretical plates (*N*), and retention factor (*K*), are within acceptable limits as exhibited in Figure 4. The system suitability tests are summarized in Table 5.

An acidic mobile phase was used in the analytical method (pH = 5) and the results of the system suitability showed good results of the tested parameters indicating the method still performs very well under the acidic pH conditions. Moreover, the method was tested at lower pH (4.9) as a part of ruggedness and robustness validation, and results were not affected by this intended lowering of the mobile phase pH.

### 3.4. *In Vitro* Hydrolysis of Codrug

**Figure 5:**
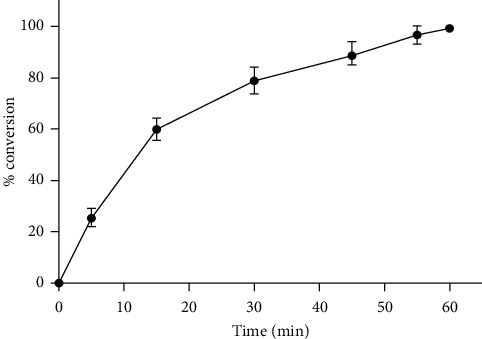
*In vitro* hydrolysis of codrug in the presence of esterase enzyme (1 U/mL).

The developed analytical method was applied to investigate the *in vitro* conversion of the codrug to its parent drugs (IND and PAR) in the presence of esterase enzyme (1 U/mL) in PBS (pH 7) at 37°C. Without the esterase enzyme, the codrug is stable in PBS (pH 7) without the observation of any hydrolysis for one month. Upon the incubation with the esterase enzyme, a decrease in the codrug peak was observed with a concomitant increase of IND and PAR HPLC peaks, and this conversion was quantified according to the developed equations. The complete conversion was observed after 60 min with a half-life of 12.2 min, as shown in Figure 5.

## 4. Conclusion

In this study, IND and PAR codrug was successfully synthesized. RP-HPLC method was developed and validated according to the ICH Q2R1 and USP guidelines for the successful separation of a mixture containing four components formula: FAM, PAR, IND, and codrug. The tested parameters, including linearity, accuracy, selectivity, precision, limits of detection and quantification, and robustness, were found to be within the recommended guideline ranges. Moreover, the developed method was successfully applied to quantify the *in vitro* hydrolysis and conversion of codrug into its parent drugs. To the best of our knowledge, this is the first study that provides an easy and direct quantification method of a combination formula of different chemical compounds (FAM, PAR, IND, and codrug).

## Figures and Tables

**Table 1:** Summary of method development optimization.

Drug	Mobile phase		pH	Diluent used
	Buffer	ACN		
FAM and IND mixture	93	7	6	Diluent 1
	93	7	5	
	50	50	5.5	
	60	40	5.5 & 6	
	40	60	5.5 & 5	

Codrug	40	60	5	Diluent 1

PAR	40	60	5	Diluent 1
				Methanol
				ACN

FAM, codrug, and IND separately	40	60	5	ACN

Mixture of all drugs	40	60	5	ACN

**Table 2:** The accuracy results in the concentration range (0.08–0.12 mg/mL).

Conc. (mg/mL)		FAM	PAR	IND	Codrug

0.08	Av. area	3070661.67	1868718.67	6561098.33	3306252.67
	% RSD	0.89	1.46	0.97	0.86
	% Recovery	99.34	100.76	100.73	99.5

0.1	Av. area	4294603.67	2347628.33	7736858.33	4166430.33
	% RSD	0.64	0.67	1.42	0.59
	% Recovery	99.5	99.98	99.22	101.38

0.12	Av. area	5238679.0	2886956.67	9774300	5300362.67
	% RSD	1.23	1.79	0.36	1.24
	% Recovery	100.33	100.89	100.88	100.82

**Table 3:** The precision results at different precision levels.

		FAM	PAR	IND	Codrug

System precision	0.1 (mg/mL)				
	Av. area	4227059	2314677	7742724	4178095
	% RSD	0.80	1.66	1.30	1.64

Intraday precision	0.08 (mg/mL)				
	Av. area	3147937	1904973	6560894	3288773
	% RSD	1.66	0.70	0.97	1.33

Interday precision	0.1 (mg/mL)				
	Av. area	4204444	2317970	7736858	4098577
	% RSD	0.76	1.75	1.42	0.20

Different analyst	0.12 (mg/mL)				
	Av. area	5249199	2878872	9739513	5286421
	% RSD	0.88	0.95	0.49	1.60

**Table 4:** Results of the robustness at different variable parameters.

		FAM	PAR	IND	Codrug

The wavelength of maximal absorption (*λ*_max_)					
273 nm	Av. area	4279022	2357627	7713100	4191009
275 nm	Av. area	4225523	2324816	7797471	4109888
277 nm	Av. area	4263527	2374482	7759887	4186203
	% RSD	0.65	1.07	0.54	1.09

Mobile pH					
pH 5.1	Av. area	4353746	2280521	7609836	4089088
pH 4.9	Av. area	4355659	2247925	7803400	4177253
pH 5.0	Av. area	4225523	2324816	7797471	4109888
	% RSD	1.73	1.69	1.42	1.12

Flow rate					
Flow rate of 1.2 mL/min.	Av. area	4289108	2336035	7620148	4220573
Flow rate of 1.4 mL/min.	Av. area	4225523	2324816	7797471	4109888
	% RSD	1.06	0.34	1.63	1.88

**Table 5:** System suitability.

	FAM	PAR	IND	Codrug

Resolution (*R*)	1.2	6.8	6.7	7.5
Symmetry of the peaks	1.1	0.9	1.1	1
Theoretical plates (*N*)	1418	2101	2160	6499
Retention factor (*K*)	1.67	2.08	5.41	10.42

## Data Availability

The data used to support the findings of this study are included within the article.
